# Arthroscopic debridement of anterior ankle impingement in patients with chronic lateral ankle instability

**DOI:** 10.1186/s12891-018-2168-6

**Published:** 2018-07-19

**Authors:** Qining Yang, Yongwei Zhou, Youjia Xu

**Affiliations:** 1 Department of Orthopaedics, The Second Affiliated Hospital of Soochow University, Sanxiang Road No.1055, Suzhou, 215004 Jiangsu China; 2 Department of joint orthopaedic surgery, Jinhua hospital of Zhejiang University (Jinhua municipal central hospital), Jinhua, Zhejiang 321000 People’s Republic of China; 3 Department of Orthopedics, 2nd Affiliated Hospital of Soochow University, Sanxiang Road No.1055, Suzhou, Jiangsu 215000 People’s Republic of China

**Keywords:** Ankle instability, Anterior ankle impingement, Osteophyte, Repair, Functional outcomes, ROM

## Abstract

**Background:**

The aim of this study was to determine the functional and radiological outcomes of arthroscopic treatment of anterior ankle impingement (AAI) in patients with chronic lateral ankle instability (CAI).

**Methods:**

All patients with CAI between June 2012 and May 2015 were invited to participate in this investigation. All of them accepted open modified Broström repair of lateral ankle ligaments and were divided into two groups: AAI group (with anterior ankle impingement) and pure CAI group (without anterior ankle impingement). All of them were followed up using American Orthopaedic Foot and Ankle Society Score (AOFAS), Karlsson Ankle Functional Score and Tegner activity score. Ankle dorsiflexion was also examined. X-ray examination was applied to investigate anterior tibiotalar osteophytes.

**Results:**

Finally, a total of 60 patients were followed up at a mean of 37 ± 10 months, including 22 patients in the AAI group and 38 patients in the pure CAI group. Preoperatively, the AAI group had significant lower AOFAS score (62.9 ± 11.7 vs 72.9 ± 11.1; *p* = 0.002) and Tegner activity score (1.5 ± 0.8 vs 2.1 ± 1.0; *p* = 0.04) respectively when compared with the pure CAI group. The ankle dorsiflexion of the AAI group (13 ± 2.1) was also significantly lower than that of the pure CAI group (26.2 ± 2.1) (*p* = 0.001). However, there was no significant difference in the AOFAS score or the Karlsson score or the Tegner score or the Ankle dorsiflexion between the two groups postoperatively. The postoperative X-ray images demonstrated complete osteophyte resection in all patients, and no recurrence of osteophyte.

**Conclusion:**

The functional outcome scores and dorsiflexion had significantly improved postoperatively. Combined treatment of chronic ankle instability and anterior ankle impingement produced satisfactory surgical outcomes in patients with CAI accompanied by anterior ankle impingement symptom.

## Background

Lateral ankle sprains occur most commonly in both sports and daily activities, and usually result in the ruptures of anterior talofibular ligament (ATFL) and calcaneofibular ligament (CFL) [1]. Although with some conservative treatment, a few injured ankles will still be unstable and have repeated sprains [2]. In ankles with chronic ankle instability (CAI), it is common to observe ankle impingement with soft tissue or osteophyte [3–6]. Previously, Odak et al. [4] reported that 63% patients had soft tissue impingement and 12% patients had anterior bony (osteophyte) impingement in a cohort of 100 CAI patients. Similarly, Hua et al. [5] reported an incidence of 86% CAI patients with soft tissue impingement and 26% CAI patients with bony impingement. Thus, it can be seen that anterior ankle impingement (AAI) had a high correlation with CAI.

According to a previous report [7], ankle impingement in anterior tibiotalar joint is caused by either mechanical macrotrauma or recurrent microtrauma associated with CAI. In particular, CAI is associated with the formation in medial ankle osteophyte, which originates from distal tibia protruding to joint line [8, 9]. Anterior ankle joint capsule is just located at proximal end of the anterior talotibial osteophyte originate [10]. In the medial part of anterior talus, osteophyte development occurs in an intraarticular location [11].

In particular, anterior tibiotalar osteophyte is a headache problem that causes severe AAI syndrome, leading to anterior ankle pain or reduction of dorsiflexion [6, 7]. Pure conservative treatment cannot easily treat the persisting bony impingement syndrome [12]. However, anterior soft tissue and bony impingement can be effectively treated arthroscopically [7, 13, 14]. After surgical debridement, the ankle function can be improved thus to allow an early return to sports activity [15]. Previously, Walsh et al. [16] evaluated functional and radiological outcomes after arthroscopic treatment of anterior ankle impingement, and demonstrated a significant improvement in the functional outcome scores (Foot Functional Index) postoperatively despite a recurrence of radiographic osteophyte. To date, there has been a lack of clinical research investigating the clinical effect of AAI debridement in CAI patients.

Therefore, the aim of this study was to investigate the functional and radiological outcomes after arthroscopic treatment of anterior ankle impingement (AAI) in patients with mechanical chronic lateral ankle instability.

## Methods

This retrospective study was approved by the Health Sciences Institutional Review Committee of Jinhua Hospital of Zhejiang University, and all participants signed a written consent form. All patients who underwent modified Broström-Gould to repair the lateral ankle ligament from June 2012 to May 2015 were required to participate in the study. Clinical diagnosis of CAI is according to a clinical history (pain or concession, repetitive varus sprain for more than 3 months), physical examination (anterior drawer test (ADT)) and MRI. Inclusion criteria were: (1) modified Broström-Gould to repair the lateral ankle ligament, and (2) follow-up for at least 2 years. Patients should be excluded if they had (1) significant cartilage lesions undergoing microfracture or autologous osteochondral transplantation, (2) prior surgery, (3) neuromuscular disease, and (4) lateral ligament reconstruction.

All patients underwent direct ATFL/CFL repair and debridement of bone and soft tissue under arthroscopy in the event of a anterior impingement. Patients underwent standard arthroscopy to evaluate the ankle joint before lateral ligament repair. Assessment and treatment of intra-articular lesions, debridement or microfracture of cartilage damage, removal of impacted soft tissue or synovial membrane, and removal of loose body. For anterior tibial impingement, the anterior lateral and anterior medial arthroscopy portals were used to remove the anterior osteophytes on the distal tibia and anterior lip of the talus. The ablation is completed until the ankle joint dorsiflexion recovered.

Then, the ATFL / CFL is repaired using the open modified Broström program according to the previous method [5]. After making a curved incision on the lateral side, the ATFL residue was identified and exposed. One or two suture anchors were inserted into the fibular (2.9-mm Loopine [DePuy Mitek, Raynham, MA]). The ankle was slightly everted. The ATFL is sutured, tensioned, and fixed onto the fibular. The proximal extensor support band is also used to strengthen the repair of the lateral ligament. All patients followed the same postoperative rehabilitation protocol. After the operation, the ankle was fixed in a slightly eversion position by using a casting. Rehabilitation training was started from the second day after surgery, including isometric contraction of muscle groups around the ankle joint, and weight bearing was allowed after 4 weeks. The casting was removed 4 weeks after the operation and changed to a sacral stent, and the range of passive motion was started.

At least 24 months after surgery, all patients were invited to this follow-up during outpatient service. Functional scores were assessed preoperatively and postoperatively, including American Orthopaedic Foot and Ankle Society (AOFAS), Karlsson Ankle Functional Score and Tegner activity score. Physical examinations, including ankle dorsiflexion and anterior drawer test (ADT), were also performed by an experienced orthopedic surgeon. The X-ray examination (lateral view) was performed to investigate the anterior tibiotalar osteophyte. Scranton and McDermott classification (SMC) grade was used to assess the osteophytes: Grade 1, tibial spur less than 3 mm; Grade 2, tibial spur more than 3 mm; Grade 3, significant tibial exostosis with secondaryspur on the neck of the talus, with eventual fragmentation of the osteophytes; Grade 4, osteophytes associated with arthritic joint destruction [16].

Statistical analysis was performed using Stata 10.0 software (Stata Corp, USA), and the data are reported as means and standard deviations. The categorical variables were compared using a χ^2^ test, and the continuous variables were compared using a two-sample t test or two-sample Wilcoxon rank-sum test. A paired students t-test was performed to compare the results between pre-op and post-op within each group. The correlation between the functional scores and various factors (gender, age, BMI, injury time, follow-up time) was analyzed using Spearman’s correlation coefficient. The significance level was set at 0.05.

## Results

A total of 60 Broström-Gould repair patients participated in this study. Among them, 22 patients had anterior ankle impingement (AAI group) and the other 38 patients had no impingement (pure CAI group). In the AAI group, all the patients had synovitis and 18 patients had osteophytes in the anterior ankle compartment. Preoperative ankle X-ray images revealed a mean SMC grade of 2 (range 1–3). Actually, most patients had only ATFL tear. Three patients (14%) in the AAI group and 5 patients (13%) in the pure CAI group had both ATFL and CFL tears. Moreover, five patients in the AAI group and 8 patients in the pure CAI group had the associated cartilage lesions. These cartilage lesions were only with a rough surface or fibrillation (Grade I), and only debridement was performed. The Patients’ demographic data was shown in Table 1. The average follow-up time was 37 months for both of the groups.

**Table 1:** Participant demographic data between the anterior ankle impingement (AAI) group and pure CAI group

	AAI group (*n* = 22)	pure CAI group (*n* = 38)
Age, mean SD, y	31.2 ± 8.8	27.6 ± 8.5 ^n.s.^
Body mass index (kg/m^2^)	24.5 ± 3.0	23.2 ± 2.4 ^n.s.^
Gender, n	Males, *n* = 16	Males, *n* = 31
	Females, *n* = 6	Females, *n* = 7
Side, n	Left, *n* = 12	Left, *n* = 15
	Right, *n* = 10	Right, *n* = 23
Injury time, months	37 ± 42	26 ± 36 ^n.s.^
Follow-up time, months	37 ± 10 (24 ~ 56)	37 ± 10 (24 ~ 53) ^n.s.^

*AAI* anterior ankle impingement. n.s. indicated there was no significant difference between groups

**Fig. 1:**
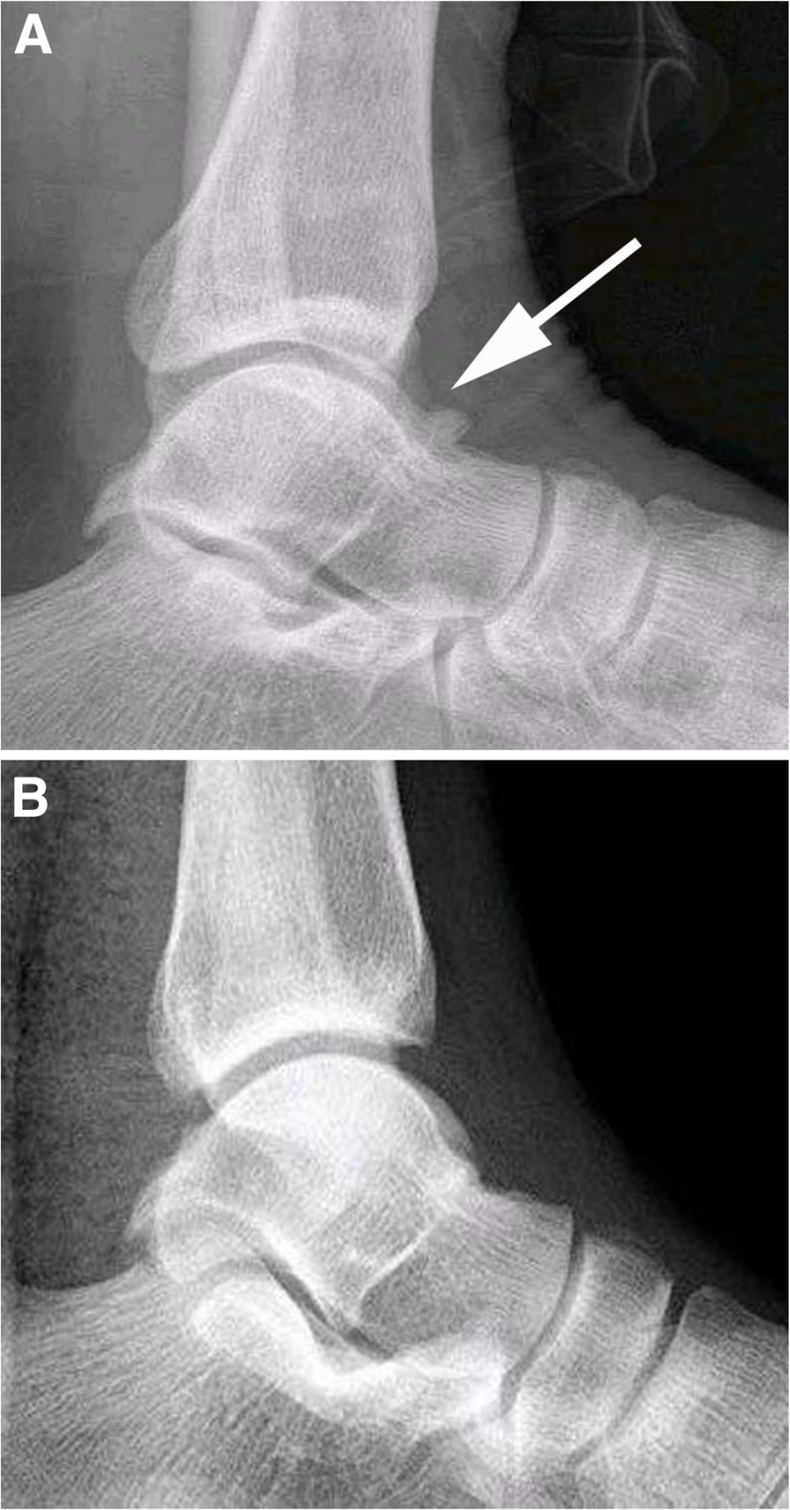
X-ray images of an ankle with bony anterior ankle impingement preoperatively (**a**) and postoperatively (**b**). White arrow indicates osteophytes

At final follow-up, no patient complained ankle instability, and ADT of all the ankles revealed negative. All these patients return to previous activity. After surgery, both groups had an increase of the functional scores. Preoperatively, the AAI group had a significant lower AOFAS score (62.9 ± 11.7 vs 72.9 ± 11.1; *p* = 0.002) and Tegner activity score (1.5 ± 0.8 vs 2.1 ± 1.0; *p* = 0.04) respectively when compared with the pure CAI group. The ankle dorsiflexion of the AAI group (13 ± 2.1°) was also significantly lower than that of the pure CAI group (26.2 ± 2.1°) (*p* = 0.001). After surgery, each functional score (AOFAS, Karlsson, Tegner activity) significantly increased (*p* < 0.05). The ankle dorsiflexion of the AAI group significantly increased from 13 ± 2.1° preoperatively to 25.9 ± 2.4° postoperatively (*p* < 0.001). There was no significant change of ankle dorsiflexion in the pure CAI group after surgery. At the final follow-up, there was no significant difference in postoperative AOFAS or Karlsson score or Tegner score or Ankle dorsiflexion between the two groups (Table 2). The postoperative X-ray images showed complete removal of the osteophytes in all patients (Fig. 1). No recurrence of osteophytes was found. There was also no statistically significant association between functional scores (AOFAS, Karlsson score, or Tegner score) and gender, age, BMI, injury time, and follow-up time (*p* > 0.05).

**Table 2:** Functional scores and Ankle dorsiflexion between the anterior ankle impingement (AAI) group and the pure CAI group pre-operatively and post-operatively

Variable		AAI group (*n* = 22)	pure CAI group (*n* = 38)	*P* value
AOFAS score	Pre-op	62.9 ± 11.7	72.9 ± 11.1	0.002
	Post-op	93.4 ± 7.4	92.3 ± 9.4	n.s.
Karlsson Score	Pre-op	57.3 ± 12.4	63.1 ± 16.5	n.s.
	Post-op	90.0 ± 9.1	89.6 ± 12.5	n.s.
Tegner score	Pre-op	1.5 ± 0.8	2.1 ± 1.0	0.04
	Post-op	4.4 ± 1.3	5.2 ± 1.8	n.s.
Ankle dorsiflexion ( °)	Pre-op	13 ± 2.1	26.2 ± 2.1	< 0.001
	Post-op	25.9 ± 2.4	26 ± 2.1	n.s.

*AAI* anterior ankle impingement, *AOFAS* American Orthopaedic Foot and Ankle Society; *Pre-op* pre-operatively, *Post-op* post-operatively “*p* value” indicated the comparison between the AAI group and the pure CAI group

Regarding the complications, one patient in the AAI group and two patients in the pure CAI group presented numbness. They recovered within one year after surgery. At two weeks after surgery, superficial wound infection was observed in 1 patients in the AAI group and 2 patients in the pure CAI group. With anti-infective treatment, these three cases recovered at one months after surgery.

## Discussion

In this study, it was found that the AAI group had a comparable results with the pure CAI group at a mean follow-up of three years. Furthermore, the postoperative X-ray images demonstrated complete osteophyte removal in all patients, and no recurrence of osteophyte was observed.

Combined lateral ligament repair and anterior ankle impingement treatment are safe and allow most patients to resume exercise [17]. Murawski et al. [18] investigated 41 patients who underwent arthroscopic treatment of AAI, and found that 38 patients (93%) were satisfied with the resection procedure. The AOFAS score increased from 62.83 points preoperatively to 91.17 points postoperatively. In the present study, most patients had osteophytes in anterior ankle compartment. It is indicated that the osteophytes were the main cause of decreased dorsiflexion. After surgical removal of the impinged osteophytes and soft tissue, patients obtained an increase of functional scores.

In the present study, the ankle dorsiflexion of the AAI group significantly increased from 13 ± 2.1° preoperatively to 25.9 ± 2.4° postoperatively. Previously, Cannon and Hackney [19] investigated 13 CAI patients with AAI and found that mean improvement of dorsiflexion was 12.4°. Previously, Ahn et al. [20] investigated 20 CAI patients with AAI, and the mean dorsiflexion improvement was 7.9 degrees in the impingement group at a mean follow-up of 1.4 years. In the present study, the mean improvement of dorsiflexion was almost 12 degrees. It was presumed that different dorsiflexion improvement might be due to different severity of osteophytes.

In this study, all the patients in the AAI group underwent open modified Broström procedure to repair ATFL and CFL. At the final follow-up, the ankle stability of all the patients recovered. It is well known that removal of the anterior osteophytes may reduce tibial coverage of talus [21]. The patient developed an impingement again after debridement alone if without ankle instability. Therefore, it is very important to ensure that the ankle joint is stable to avoid micro-trauma of the tibialtalar joint. ATFL and CFL repair using suture anchors is an effective method for the treatment of CAI [22]. In addition, it has recently been reported that arthroscopic repair of the lateral ankle ligament may produce similar beneficial results compared with open ankle repair [23]. Arthroscopic ATFL repair as a minimally invasive technique may provide more favorable results.

Previously, Walsh et al. [16] assessed functional and radiological outcomes after arthroscopic treatment of AAI, and reported a recurrence of radiological osteophyte in 84% of patients. In the present study with a mean follow-up of 3 years, no recurrence of osteophyte was found and the postoperative X-ray images showed complete osteophyte resection in all patients. The difference might be due to all the patients had ankle stability after repair surgery in the present study, while the patients only accepted debridement of ankle impingement in that study by Walsh et al. [16].

There are three limitations that need to be addressed. The first limitation is the small sample size of the AAI group and the pure CAI group. Other studies with larger sample sizes may be needed to determine differences in clinical outcomes between groups. The study documented short-term results and more research was needed to determine long-term outcomes for this patient population. In addition, there was a lack of comparison in the AAI group without surgery or placebo.

## Conclusions

Functional outcome scores and dorsiflexion were significantly improved after 3 years of ligament repair and impingement treatment. Comprehensive treatment of CAI and AAI produces satisfactory clinical results in patients with CAI associated with anterior impingement symptoms.

### Level of evidence

level III. Cohort study.

## Abbreviations

AAI: Anterior ankle instability
ADT: Anterior drawer test
AOFAS: American Orthopaedic Foot and Ankle Society
ATFL: Anterior talofibular ligament
CAI: Chronic ankle instability
CFL: Calcaneofibular ligament
